# Pretreatment C‐reactive protein/albumin ratio is associated with poor survival in patients with stage IB‐IIA cervical cancer

**DOI:** 10.1002/cam4.1270

**Published:** 2017-11-28

**Authors:** Weiwei Zhang, Kejun Liu, Bin Ye, Weijiang Liang, Yazhou Ren

**Affiliations:** ^1^ Department of Medical Oncology The Sixth People's Hospital of Chengdu 610051 Sichuan China; ^2^ Department of Medical Oncology Dongguan People's Hospital 523059 Guangdong China; ^3^ Department of Medical Oncology Nanfang Hospital Southern Medical University 510515 Guangdong China; ^4^ Big Data Research Center School of Computer Science and Engineering University of Electronic Science and Technology of China 611731 Sichuan China

**Keywords:** Cervical cancer, C‐reactive protein/albumin ratio, predictor of survival

## Abstract

Previous studies have shown that the C‐reactive protein/albumin ratio (CAR) is a prognostic indicator in multiple types of carcinomas. This study is the first to evaluate the prognostic significance of CAR in stage IB‐IIA cervical cancer patients treated with radical surgery, as well as that of several other inflammation‐based factors, including the neutrophil‐to‐lymphocyte ratio (NLR), platelet‐to‐lymphocyte ratio (PLR), and prognostic nutritional index (PNI). A total of 235 patients were enrolled in this study. The optimal cut‐off values of CAR and other inflammation‐based factors were determined by receiver operating characteristic curves. The Kaplan–Meier method and Cox regression model analysis were performed to determine the independent predictors of progression‐free survival (PFS) and overall survival (OS). At a cut‐off value of 0.15, patients with a high CAR had significantly shorter PFS and OS than those with a lower CAR (*P *< 0.001). A higher CAR was significantly associated with elevated scores of NLR and PLR and a decreased PNI (*P *< 0.001). Univariate analyses showed that elevated CAR preoperatively was significantly associated with poor survival; a similar trend was also noted for the NLR, PLR, and PNI. Multivariate analyses demonstrated that only CAR was an independent indicator for PFS (hazard ratio [HR]: 5.164; 95% confidence interval [CI]: 2.495–10.687; *P *< 0.001) and OS (HR: 4.729; 95% CI: 2.263–9.882; *P *< 0.001). In conclusion, preoperative CAR is a novel and superior predictor of poor survival in patients with stage IB‐IIA cervical cancer.

## Introduction

Cervical cancer is one of the most common cancers worldwide and is the primary cause of cancer‐related deaths in women 1. In 2012, approximately 527,600 new cervical cancer patients were diagnosed, and 265,700 deaths occurred worldwide 2. Cervical cancer is currently divided into two different tumor types, squamous cell carcinoma and nonsquamous cell carcinoma. Approximately 80% of cases are squamous cell carcinoma, and the remaining 20% belong to the adenocarcinoma subgroup. For patients with IB‐IIA stage cervical cancer, radical hysterectomy followed by chemotherapy or chemoradiation are effective treatment options 3. Nevertheless, once tumor recurrence occurs, patients with cervical cancer will have a poor prognosis because of limited therapeutic strategies in the clinic 4, 5. To date, there are no well‐established prognostic laboratory biomarkers that can better predict the recurrence and clinical outcomes of cervical cancer patients, except for the squamous cell carcinoma antigen 6. Therefore, finding viable and reliable prognostic biomarkers is urgently needed to develop individualized therapy in the clinic.

Various cancer‐related inflammatory mediators can promote tumor growth, invasion, metastasis, and angiogenesis after induction by inflammatory or tumor cells 6, 7. Human papillomavirus (HPV) can promote the development of cervical cancer by triggering a series inflammation and inflammatory molecules in the microenvironment 8. It was previously shown that one way to improve the nutritional status is to attenuate the systemic inflammatory response 9. Basic and clinical research results have suggested that hypoalbuminemia, malnutrition, and cancer cachexia are all consequences of the body's systemic inflammatory response to malignancy 10. Zheng et al. also showed that albumin is an independent prognostic indicator in early operable cervical cancer 7. Recently, several inflammation‐ and nutrition‐based scores such as the platelet‐to‐lymphocyte ratio (PLR), neutrophil‐to‐lymphocyte ratio (NLR), and prognostic nutritional index (PNI) have been demonstrated as significant predictors in cervical cancer as well as other cancers 11, 12, 13, 14. The PNI is calculated according to the following formula: serum albumin value (g/L) + 0.005 × lymphocyte counts (per mm³) in peripheral blood 14. However, the most common measure of the systemic inflammatory response in cancer patients is the serum concentration of C‐reactive protein (CRP), which can identify patients who will likely develop cachexia and have a poor survival and response to treatment after combining with albumin to a scale 9, 15. As a novel inflammation‐ and nutrition‐based score, the CRP/albumin ratio (CAR) is a combination of CRP and albumin. It has been reported as an independent indicator of poor prognosis in different malignant carcinomas including hepatic cancer 16, lung cancer 17, colon cancer 18, esophageal cancer 19, and pancreatic cancer 20. Currently, there are no reports concerning CAR as a predictor in patients with cervical cancer. Hence, we retrospectively studied the intrinsic tumor factors along with inflammation‐based scores to identify superior prognostic factors in cervical cancer patients with stage IB‐IIA disease.

## Methods

### Patients

A total of 235 IB‐IIA cervical cancer patients who had radical hysterectomy and pelvic lymphadenectomy at Nanfang Hospital of Southern Medical University from January 2005 to December 2009 were included in this study. Patients who had an active or chronic infection received chemotherapy or radiotherapy before surgery, hematological and autoimmune disorders, or did not have available follow‐up data were excluded. Patients whose clinical information could not be completely obtained were also excluded from our analysis. The tumor stage of all cases was defined according to the FIGO 2009 criteria for cervical cancer. Clinical data including the treatment programs (surgery, chemoradiotherapy, or radiotherapy), maximal tumor size, and histopathological parameters were retrospectively achieved from the medical data of cervical cancer patients. Laboratory parameters including the neutrophil, lymphocyte, and platelet counts, and CRP and albumin levels were obtained within 2 days before radical surgery. This study was approved by the medical ethics committee of Nanfang Hospital of Southern Medical University. All the methods were performed in accordance with the relevant guidelines and regulations. Written informed consent was obtained for each patient.

### Clinical management

All the patients with FIGO stage IB‐IIA cervical cancer were managed by radical hysterectomy and pelvic lymphadenectomy. Para‐aortic lymph node dissection was performed in patients who had suspicious para‐aortic lymph node metastasis. Postoperative radiotherapy with or without concurrent platinum‐based chemotherapy was treated depending on the postoperative pathology. PFS was defined as the time from the start of surgery to recurrence or death. OS was defined as the time from the start of the surgery to the last contact or death. All of the cervical cancer patients were followed up every 3 months for the first 2 years, every 6 months for the next 5 years, and annually thereafter until January 2014. Gynecological examinations, laboratory checks, and imaging methods (e.g., head and abdominal ultrasound scanning, chest X‐ray, electrocardiography, and computed tomography) were conducted during follow‐up evaluations.

### Statistical analysis

Statistical analyses were performed using Statistical Product and Service Solutions 20.0 software (IBM, Corporation, Armonk, NY). The best cutoff points of NLR, PLR, PNI, and CAR were determined using receiver operating characteristic (ROC) curve analysis. The relationships between CAR and other variables were performed using Pearson's chi‐square test. Significant prognostic variables in univariate analyses were selected for multivariate Cox regression model analyses to determine independent prognostic factors using the forward stepwise method. Estimates of PFS and OS among the classification groups were calculated using the Kaplan–Meier method, and two‐sided 95% confidence intervals were obtained. A two‐sided *P* < 0.05 was considered statistically significant.

## Results

**Figure 1:**
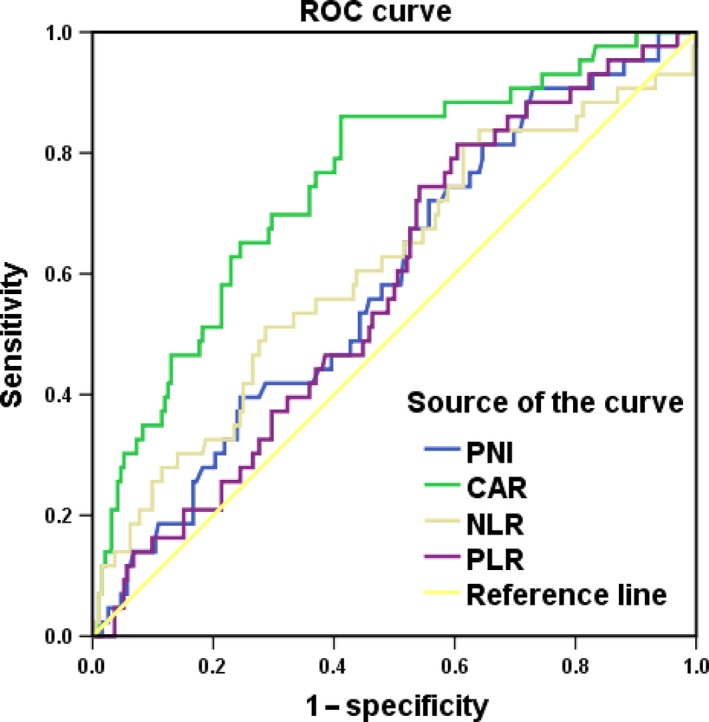
Receiver operating characteristic curves for the PNI, CAR, NLR, and PLR for OS.

The number of eligible patients in our study was 235. The clinical characteristics of these patients are listed in Table 1. The median age at the time of diagnosis was 46 years (range: 29–78 years). The median follow‐up time was 77 months (range: 32–96 months). Among these patients, 122 (51.9%) were FIGO stage IB, and the remaining 113 (48.1%) were stage IIA. The numbers of patients with a maximum tumor size ≤4 cm and >4 cm were 152 (64.7%) and 83 (35.3%), respectively. According to the ROC curves, the best cut‐off points for the CAR, NLR, PNI, and PLR were 0.15, 4.0, 50.38, and 176.5, respectively, corresponding to maximum joint sensitivity and specificity. For the CAR, NLR, PLR, and PNI, the areas under the ROC curve for OS were 0.751, 0.611, 0.577, and 0.588, respectively, and the sensitivities (specificities) were 79.1% (59.4%), 51.2% (71.4%), 81.4% (39.6%), and 90.7% (27.1%), respectively (Fig. 1). The relationship between the preoperative CAR and clinicopathological parameters of cervical cancer patients is shown in Table 1. Patients with a high CAR (>0.15) were more prone to have a lower PNI, and higher NLR and PLR (all *P *< 0.001). However, there was no significant difference between a high CAR and age (*P *= 0.978), tumor stage (*P *= 0.139), tumor size (*P *= 0.264), pathological type (*P *= 0.242), adjuvant therapy (*P *= 0.748), tumor grade (*P *= 0.406), lymphovascular space invasion (*P *= 0.524), lymphatic metastasis (*P *= 0.521), or depth of invasion (*P *= 0.946).

**Table 1:** Correlations between preoperative CAR and clinicopathological characteristics

Variable	Cases (*n* = 235)	CAR ≤ 0.15 (*n* = 122)	CAR > 0.15 (*n* = 113)	*P*‐value
Age				
≤46 years	125 (53.2)	65 (53.3)	60 (53.1)	0.978
>46 years	110 (46.8)	57 (46.7)	53 (46.9)	
Tumor stage				
IB	122 (51.9)	69 (56.6)	53 (46.9)	0.139
IIA	113 (48.1)	53 (43.4)	60 (53.1)	
Maximum tumor size				
≤4.0 cm	152 (64.7)	83 (68.0)	69 (61.1)	0.264
>4.0 cm	83 (35.3)	39 (32.0)	44 (38.9)	
Pathological type				
Squamous	225 (95.7)	115 (94.3)	110 (97.3)	0.242
Nonsquamous	10 (4.3)	7 (5.7)	3 (2.7)	
Adjuvant therapy				
None	51 (21.7)	25 (20.5)	26 (23.0)	0.748
Chemoradiotherapy	137 (58.3)	74 (60.7)	63 (55.8)	
Radiotherapy	47 (20.0)	23 (18.8)	24 (21.2)	
Tumor grade				
G1	49 (20.8)	29 (23.8)	20 (17.7)	0.406
G2	144 (61.3)	74 (60.6)	70 (61.9)	
G3	42 (17.9)	19 (15.6)	23 (20.4)	
Lymphovascular space invasion				
No	213 (90.6)	112 (91.8)	101 (89.4)	0.524
Yes	22 (9.4)	10 (8.2)	12 (10.6)	
Lymphatic metastasis				
Negative	198 (84.3)	101 (82.8)	97 (85.8)	0.521
Positive	37 (15.7)	21 (17.2)	16 (14.2)	
Depth of invasion				
<2/3	117 (49.8)	61 (50.0)	56 (49.6)	0.946
≥2/3	118 (50.2)	61 (50.0)	57 (50.4)	
NLR				
≤4.0	158 (67.2)	110 (90.2)	48 (42.5)	<0.001
>4.0	77 (32.8)	12 (9.8)	65 (57.5)	
PLR				
≤176.5	84 (35.7)	64 (52.5)	20 (17.7)	<0.001
>176.5	151 (64.3)	58 (47.5)	93 (82.3)	
PNI				
≤50.38	179 (76.2)	76 (62.3)	103 (91.2)	<0.001
>50.38	56 (23.8)	46 (37.7)	10 (8.8)	

CAR, C‐reactive protein/albumin ratio; NLR, neutrophil‐to‐lymphocyte ratio; PLR, platelet‐to‐lymphocyte ratio; PNI, prognostic nutritional index.

**Figure 2:**
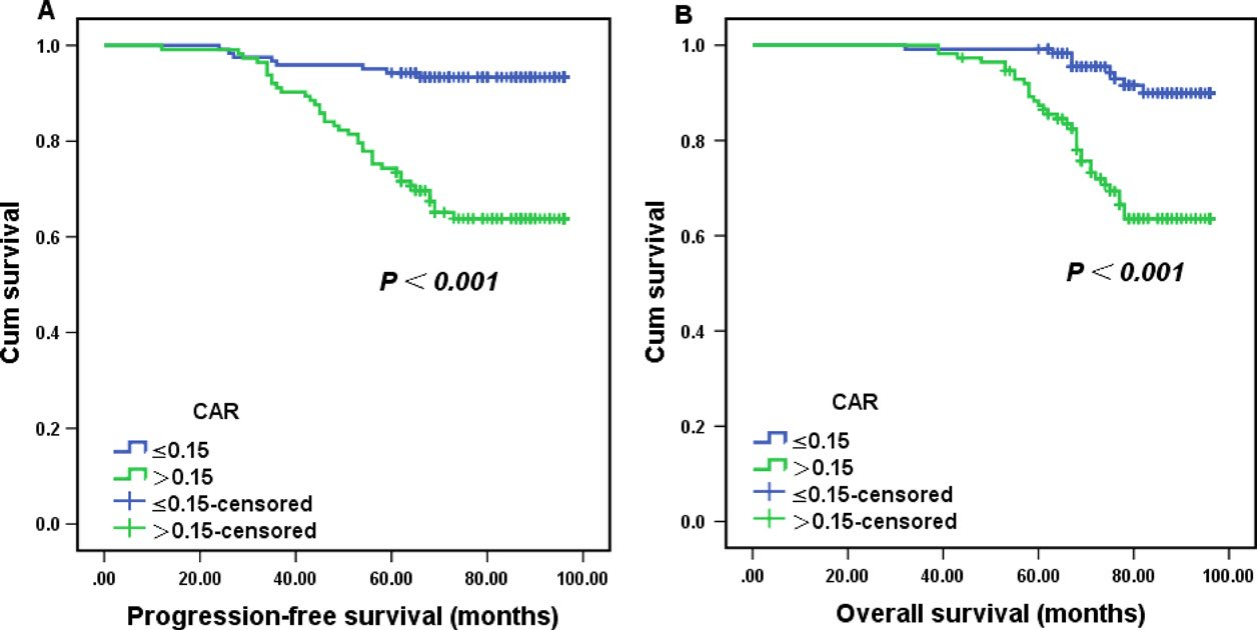
Kaplan–Meier survival curves showing progression‐free survival (A) and overall survival (B) according to the C‐reactive protein/albumin ratio. *P*‐values were determined using the log‐rank test.

The large patient cohort was divided into high‐CAR (>0.15) and low‐CAR (≤0.15) groups using the cut‐off value. Kaplan–Meier analysis confirmed that patients in the CAR > 0.15 group had significantly shorter PFS and OS than those in the CAR ≤ 0.15 group in stage IB‐IIA cervical cancer (all *P *< 0.001, Fig. 2). Similarly, patients with a high CAR had lower cumulative 3‐ and 5‐year PFS rates (84.1% and 65.0%, respectively) and OS rates (97.3% and 73.7%, respectively) than those in the low CAR group (PFS: 95.9% and 93.3%, respectively; all *P *< 0.001; OS: 99.2% and 95.4%, respectively; all *P *< 0.001).

Univariate and multivariate analyses were performed to demonstrate whether the CAR is an independent predictor of poor survival in cervical cancer patients. In univariate analyses, the tumor stage (*P *= 0.006), tumor size (*P *= 0.028), adjuvant therapy (*P *= 0.030), NLR (*P *= 0.002), PLR (*P *= 0.010), PNI (*P *= 0.024), and CAR (*P *< 0.001) were significantly associated with PFS (Table 2). Moreover, the tumor stage (*P *= 0.011), maximum tumor size (*P *= 0.022), adjuvant therapy (*P *= 0.039), NLR (*P *= 0.004), PLR (*P *= 0.015), PNI (*P *= 0.025), and CAR (*P *< 0.001) were significantly associated with OS (Table 3). In the multivariate Cox regression model, the tumor stage (HR: 2.122; 95% CI: 1.162–3.875; *P *= 0.014), adjuvant therapy (HR: 1.648; 95% CI: 1.035–2.623; *P *= 0.035), and CAR (HR: 5.164; 95% CI: 2.495–10.687; *P *< 0.001) were significantly associated with PFS, whereas the NLR, PLR, and PNI did not predict PFS (Table 2). In addition, the tumor stage (HR 2.073, 95% CI 1.105–3.887, *P *= 0.023), adjuvant therapy (HR 1.653, 95% CI 1.024–2.668, *P *= 0.040), and CAR (HR 4.729, 95% CI 2.263–9.882, *P *< 0.001) were also independently and significantly associated with clinical outcomes (OS) (Table 3). All these data showed that preoperative CAR was an independent indicator for predicting a poor PFS and OS in cervical cancer patients who had stage IB‐IIA stage and radical surgery.

**Table 2:** Univariate and multivariate analyses of progression‐free survival in cervical cancer patients

Variable	Univariate analyses HR (95% CI)	*P*‐value	Multivariate analyses HR (95% CI)	*P*‐value
Age				
≤46 years	1	0.562		
>46 years	1.182 (0.671–2.082)			
Tumor stage				
IB	1	0.006	1	0.014
IIA	2.340 (1.284–4.265)		2.122 (1.162–3.875)	
Maximum tumor size				
≤4.0 cm	1	0.028		
>4.0 cm	1.883 (1.069–3.315)			
Pathological type				
Squamous	1	0.428		
Nonsquamous	1.605 (0.499–5.165)			
Adjuvant therapy				
None	1	0.030	1	0.035
Chemoradiotherapy	1.629 (1.048–2.532)		1.648 (1.035–2.623)	
Radiotherapy				
Tumor grade				
G1	1	0.687		
G2	1.097 (0.700–1.719)			
G3				
Lymphovascular space invasion				
No	1	0.709		
Yes	1.193 (0.473–3.012)			
Lymphatic metastasis				
No	1	0.545		
Yes	0.768 (0.326–1.806)			
Depth of invasion				
<2/3	1	0.423		
≥2/3	1.263 (0.714–2.235)			
NLR				
≤4.0	1	0.002		
>4.0	2.435 (1.382–4.291)			
PLR				
≤176.5	1	0.010		
>176.5	2.595 (1.257–5.358)			
PNI				
≤50.38	1	0.024		
>50.38	2.898 (1.148–7.318)			
CAR				
≤0.15	1	<0.001	1	<0.001
>0.15	5.454 (2.639–11.264)		5.164 (2.495–10.687)	

CAR, C‐reactive protein/albumin ratio; NLR, neutrophil‐to‐lymphocyte ratio; PLR, platelet‐to‐lymphocyte ratio; PNI, prognostic nutritional index.

**Table 3:** Univariate and multivariate analyses of overall survival in cervical cancer patients

Variable	Univariate analyses HR (95% CI)	*P*‐value	Multivariate analyses HR (95% CI)	*P*‐value
Age				
≤46 years	1	0.735		
>46 years	1.109 (0.610–2.016)			
Tumor stage				
IB	1	0.011	1	0.023
IIA	2.262 (1.208–4.236)		2.073 (1.105–3.887)	
Maximum tumor size				
≤4.0 cm	1	0.022		
>4.0 cm	2.013 (1.106–3.662)			
Pathological type				
Squamous	1	0.391		
Nonsquamous	1.672 (0.517–5.408)			
Adjuvant therapy				
None	1	0.039	1	0.040
Chemoradiotherapy	1.620 (1.024–2.564)		1.653 (1.024–2.668)	
Radiotherapy				
Tumor grade				
G1	1	0.569		
G2	1.147 (0.716–1.838)			
G3				
Lymphovascular space invasion				
No	1	0.555		
Yes	1.324 (0.521–3.365)			
Lymphatic metastasis				
No	1	0.808		
Yes	0.899 (0.379–2.129)			
Depth of invasion				
<2/3	1	0.200		
≥2/3	1.487 (0.811–2.729)			
NLR				
≤4.0	1	0.004		
>4.0	2.422 (1.332–4.405)			
PLR				
≤176.5	1	0.015		
>176.5	2.598 (1.205–5.601)			
PNI				
≤50.38	1	0.025		
>50.38	3.239 (1.158–9.066)			
CAR				
≤0.15	1	<0.001	1	<0.001
>0.15	4.924 (2.360–10.272)		4.729 (2.263–9.882)	

CAR, C‐reactive protein/albumin ratio; NLR, neutrophil to lymphocyte ratio; PLR, platelet to lymphocyte ratio; PNI, prognostic nutritional index.

## Discussion

Chronic inflammation is involved in cellular transformation, survival, proliferation, invasion, and metastasis of tumor cells 8. As an inflammation‐sensitive marker, serum CRP has been demonstrated to be an independent prognostic indicator for clinical outcomes and posttherapy monitoring in cervical cancer 21, 22. Interleukin‐6 (IL‐6) can be released from the tumor microenvironment by leukocytes and promote the generation of CRP 23, 24. High CRP is most likely to be associated with tumor necrosis, local tissue damage, and inflammation response in cancer patients 25. A study by Polterauer et al. demonstrated that polymorphisms of the CRP gene can weaken the immune defense of tumor patients and increase the serum CRP levels 22. CRP‐lowering drugs, such as COX inhibitors and lipid‐lowering agents, have already been confirmed as effective therapeutic targets for patients undergoing cardiovascular therapy, providing promising clues for theoretical efficacy in cancer prevention and therapy 26. Many epidemiological studies have shown that the long‐term use of COX inhibitors and lipid‐lowering agents is related to decreased risk of various human cancers including cervical cancer 26, 27. Previous studies have shown that statins, CRP‐lowering drugs, have antitumor proliferative, antitumor angiogenic, and antitumor metastatic properties, and therefore, represent a novel treatment method for cancer prevention and treatment 28. Furthermore, anti‐IL‐6 therapy not only decreases CRP levels but also improves the nutritional status of cancer patients by increasing their appetite 29.

Malnutrition accounts for 20% of all cancer‐related deaths 30. Approximately 40–80% of all cancer patients will experience malnutrition at some stage during the clinical course of their disease 30. In fact, studies have demonstrated that approximately 62–88% of gynecological cancer patients will experience malnutrition 31, 32. Various factors including hemoglobin, total protein, albumin, and transferrin are used to evaluate the nutritional status in patients with gynecological cancer 33. Malnutrition and inflammation suppresses the synthesis of serum albumin, which can reflect the nutritional status of patients, as well as the severity, progression, and prognosis of disease 34. IL‐6 can regulate albumin production and decrease the serum albumin concentration 35. Similarly, the serum albumin level, which correlates well to body immunity and nutrition status, may increase the CRP concentration and induce inflammation 36, 37. Serum albumin has been shown as an independent predictor of clinical outcomes in various cancers, such as lung cancer, breast cancer, colorectal cancer, ovarian, and cervical cancer 33, 38. In addition, many studies have combined CRP and albumin to create new inflammation‐based scores such as the Glasgow Prognostic Score (GPS) and CAR. Both the GPS and CAR have been confirmed as independent indicators of poor survival in various cancer types 39, 40. To date, the relationship between CAR and cervical cancer prognosis is obscure. Hence, we decided to combine CRP and serum albumin to create a new inflammatory prognostic factor in stage IB‐IIA cervical cancer.

This study was the first to report the correlation between CAR and clinical outcomes in cervical cancer patients. We assessed and compared the prognostic value of the preoperative PNI, PLR, NLR, and CAR in 235 patients with stage IB‐IIA cervical cancer by retrospectively analyzing the clinical characteristics. A 0.15 cut‐off value for CAR was used to divide patients into two groups. According to the chi‐square test, the CAR had a significant relationship with other inflammation‐based scores, but no significant relationship with all of the intrinsic factors. When patients were stratified into low‐CAR and high‐CAR subgroups, patients with a higher CAR had a significantly shorter PFS and OS than those with a lower CAR. Univariate analyses indicated that tumor stage, maximum tumor size, adjuvant therapy, and all of the inflammation‐based scores (PLR, NLR, PNI, and CAR) were significantly related to a poor PFS and OS in cervical cancer patients of stage IB‐IIA. Multivariate analyses demonstrated that only tumor stage, adjuvant therapy, and CAR were independent prognostic factors for predicting poor PFS and OS in cervical cancer patients.

However, this study had several limitations. First, it was inevitably affected by residual confounding factors. Second, we only included stage IB‐IIA cervical cancer patients. Third, the number of patients was relatively small. Finally, it was single retrospective study.

In conclusion, the preoperative CAR is a novel and superior prognostic factor in cervical cancer patients with stage IB‐IIA and radical surgery. More clinical trials are needed to further elaborate upon the relationship between CAR and the prognosis of stage IB‐IIA cervical cancer.

## Conflict of Interest

None declared.

## Supporting information


**Table S1.** The original data for lymphocyte, albumin, and PNI.
