# Corrigendum

**DOI:** 10.1002/cam4.1599

**Published:** 2018-06-20

Pretreatment C‐reactive protein/albumin ratio is associated with poor survival in patients with stage IB‐IIA cervical cancer

Weiwei Zhang, Kejun Liu, Bin Ye, Weijiang Liang & Yazhou Ren

Cancer Medicine 2018; 7: 105‐113.

The first author of this article is also affiliated to “Department of Medical Oncology, Nanfang Hospital, Southern Medical University, Guangzhou 510515, China” and this should be the Dr. Zhang’s first affiliation. Please see below author byline and their correct affiliation:

**Weiwei Zhang** ^**1**,2^, **Kejun Liu** ^3^, **Bin Ye** ^2^, **Weijiang Liang** ^1^ & **Yazhou Ren** ^4^

^1^Department of Medical Oncology, Nanfang Hospital, Southern Medical University, 510515 Guangdong, China

^2^Department of Medical Oncology, The Sixth People’s Hospital of Chengdu, 610051 Sichuan, China

^3^Department of Medical Oncology, Dongguan People’s Hospital, 523059 Guangdong, China

^4^Big Data Research Center, School of Computer Science and Engineering, University of Electronic Science and Technology of China, 611731 Sichuan, China

Additionally, Dr. Weijiang Liang (email: wjliang22@126.com) is also the corresponding author of this article and this paper is funded by Guangdong Province Science and Technological Program in China (Grant no. 2011B031800042).

The corresponding author apologises for this error.

Zhang W, Liu K, Ye B, Liang W, Ren Y. Pretreatment C‐reactive protein/albumin ratio is associated with poor survival in patients with stage IB‐IIA cervical cancer. *Cancer Med*. 2018;7:105:113.

