# Supplementary material for: Pretreatment C‐reactive protein/albumin ratio is associated with poor survival in patients with stage IB‐IIA cervical cancer
Source: Cancer Med. 2017 Nov 28;7(1):105–13. doi: 10.1002/cam4.1270 (PMC5773960; doi:10.1002/cam4.1270)
Supplement: Supplementary file 1 — Table S1. The original data for lymphocyte, albumin, and PNI. [file CAM4-7-105-s001.docx]

Table 1 The original data for lymphocyte, albumin and PNI.

| Patients | Lymphocyte (x10^9^/L) | Albumin (g/L) | PNI |
| --- | --- | --- | --- |
| 1 | 1.63 | 43.6 | 51.75 |
| 2 | 2.37 | 34 | 45.85 |
| 3 | 2.53 | 40 | 52.65 |
| 4 | 0.89 | 39.9 | 44.35 |
| 5 | 0.56 | 27.4 | 30.2 |
| 6 | 1.23 | 41 | 47.15 |
| 7 | 0.52 | 41.5 | 44.1 |
| 8 | 1.05 | 52.2 | 57.45 |
| 9 | 0.63 | 36.6 | 39.75 |
| 10 | 1.43 | 38.6 | 45.75 |
| 11 | 2.38 | 35.4 | 47.3 |
| 12 | 2.35 | 36.6 | 48.35 |
| 13 | 1.58 | 39.3 | 47.2 |
| 14 | 1.87 | 44.4 | 53.75 |
| 15 | 1.36 | 39.8 | 46.6 |
| 16 | 2.23 | 42 | 53.15 |
| 17 | 0.44 | 31.2 | 33.4 |
| 18 | 0.52 | 29 | 31.6 |
| 19 | 1.59 | 37.2 | 45.15 |
| 20 | 3.72 | 39.1 | 57.7 |
| 21 | 2.19 | 43 | 53.95 |
| 22 | 1.08 | 42.7 | 48.1 |
| 23 | 1.59 | 39.4 | 47.35 |
| 24 | 1.57 | 42.7 | 50.55 |
| 25 | 1.78 | 44.5 | 53.4 |
| 26 | 1.17 | 41.8 | 47.65 |
| 27 | 1.05 | 47.2 | 52.45 |
| 28 | 1.66 | 38.7 | 47 |
| 29 | 1.84 | 43 | 52.2 |
| 30 | 0.76 | 40.8 | 44.6 |
| 31 | 1.65 | 41.6 | 49.85 |
| 32 | 3.14 | 44.5 | 60.2 |
| 33 | 1.54 | 38.6 | 46.3 |
| 34 | 1.87 | 37 | 46.35 |
| 35 | 1.75 | 39.7 | 48.45 |
| 36 | 2.07 | 41.1 | 51.45 |
| 37 | 0.62 | 46.9 | 50 |
| 38 | 1.67 | 43.1 | 51.45 |
| 39 | 0.91 | 32.9 | 37.45 |
| 40 | 2.47 | 45.5 | 57.85 |
| 41 | 1.24 | 23.4 | 29.6 |
| 42 | 0.95 | 39 | 43.75 |
| 43 | 1.29 | 34.9 | 41.35 |
| 44 | 0.93 | 37.5 | 42.15 |
| 45 | 3.44 | 45.8 | 63 |
| 46 | 1.57 | 35.9 | 43.75 |
| 47 | 0.81 | 40 | 44.05 |
| 48 | 1.92 | 42.7 | 52.3 |
| 49 | 1.67 | 45.8 | 54.15 |
| 50 | 1.63 | 38.4 | 46.55 |
| 51 | 1.71 | 40.4 | 48.95 |
| 52 | 1.46 | 36 | 43.3 |
| 53 | 0.98 | 32.8 | 37.7 |
| 54 | 1.52 | 33 | 40.6 |
| 55 | 1.73 | 33 | 41.65 |
| 56 | 1.55 | 36.3 | 44.05 |
| 57 | 1.2 | 43 | 49 |
| 58 | 1.17 | 30.1 | 35.95 |
| 59 | 1.6 | 38 | 46 |
| 60 | 1.79 | 32 | 40.95 |
| 61 | 3.29 | 38.7 | 55.15 |
| 62 | 1.93 | 37.2 | 46.85 |
| 63 | 1.2 | 38.7 | 44.7 |
| 64 | 1.23 | 38.6 | 44.75 |
| 65 | 1.58 | 38.1 | 46 |
| 66 | 1.15 | 43.1 | 48.85 |
| 67 | 1.98 | 43.8 | 53.7 |
| 68 | 1.09 | 32.6 | 38.05 |
| 69 | 1.27 | 32.7 | 39.05 |
| 70 | 1.5 | 41.1 | 48.6 |
| 71 | 1.02 | 39.2 | 44.3 |
| 72 | 1.59 | 36.9 | 44.85 |
| 73 | 3.2 | 40 | 56 |
| 74 | 1.45 | 40 | 47.25 |
| 75 | 3.45 | 40 | 57.25 |
| 76 | 1.19 | 37.2 | 43.15 |
| 77 | 2.05 | 34 | 44.25 |
| 78 | 0.96 | 33.7 | 38.5 |
| 79 | 1.8 | 42.5 | 51.5 |
| 80 | 1.11 | 38.6 | 44.15 |
| 81 | 0.64 | 38 | 41.2 |
| 82 | 1.52 | 39 | 46.6 |
| 83 | 1.87 | 36.2 | 45.55 |
| 84 | 2.12 | 39.7 | 50.3 |
| 85 | 4.77 | 37.1 | 60.95 |
| 86 | 1.07 | 40 | 45.35 |
| 87 | 2.02 | 45 | 55.1 |
| 88 | 1.43 | 36.4 | 43.55 |
| 89 | 2.23 | 39 | 50.15 |
| 90 | 2.1 | 43.2 | 53.7 |
| 91 | 2.35 | 39.4 | 51.15 |
| 92 | 2 | 35 | 45 |
| 93 | 1.35 | 29 | 35.75 |
| 94 | 0.8 | 30 | 34.01 |
| 95 | 1.54 | 35 | 42.7 |
| 96 | 1.51 | 26.4 | 33.95 |
| 97 | 1.54 | 34.6 | 42.3 |
| 98 | 1.8 | 31.8 | 40.8 |
| 99 | 1.35 | 39.1 | 45.85 |
| 100 | 1.3 | 34.8 | 41.3 |
| 101 | 1.71 | 31.1 | 39.65 |
| 102 | 1.68 | 37.6 | 46 |
| 103 | 2.51 | 25.1 | 37.65 |
| 104 | 1.16 | 34 | 39.8 |
| 105 | 2 | 40.6 | 50.6 |
| 106 | 1.67 | 45.1 | 53.45 |
| 107 | 1.49 | 39 | 46.45 |
| 108 | 2.25 | 35.7 | 46.95 |
| 109 | 0.89 | 28.9 | 33.35 |
| 110 | 0.9 | 33.9 | 38.4 |
| 111 | 1.1 | 38.8 | 44.3 |
| 112 | 1.88 | 35 | 44.4 |
| 113 | 1.87 | 29.4 | 38.75 |
| 114 | 1.76 | 34.8 | 43.6 |
| 115 | 1.21 | 39.8 | 45.85 |
| 116 | 0.81 | 34.4 | 38.45 |
| 117 | 1.01 | 30.2 | 35.25 |
| 118 | 1.94 | 46.7 | 56.4 |
| 119 | 1.56 | 31.3 | 39.1 |
| 120 | 1.58 | 36.6 | 44.5 |
| 121 | 1.9 | 43 | 52.5 |
| 122 | 1.82 | 38 | 47.1 |
| 123 | 1.61 | 29.5 | 37.55 |
| 124 | 1.01 | 40.9 | 45.95 |
| 125 | 2.41 | 42.7 | 54.75 |
| 126 | 1.24 | 29.3 | 35.5 |
| 127 | 1.44 | 38.7 | 45.9 |
| 128 | 1.93 | 43.5 | 53.15 |
| 129 | 1.05 | 40.5 | 45.75 |
| 130 | 1.5 | 30.5 | 38 |
| 131 | 1.87 | 37 | 46.35 |
| 132 | 1.7 | 43 | 51.5 |
| 133 | 1.19 | 30 | 35.95 |
| 134 | 1.7 | 45.1 | 53.6 |
| 135 | 1.32 | 29.5 | 36.1 |
| 136 | 0.66 | 35.1 | 38.4 |
| 137 | 1.15 | 39 | 44.75 |
| 138 | 1.6 | 39 | 47 |
| 139 | 1.74 | 23.7 | 32.4 |
| 140 | 1.2 | 35 | 41 |
| 141 | 3.27 | 41.4 | 57.75 |
| 142 | 2.25 | 32 | 43.25 |
| 143 | 1.72 | 43.6 | 52.2 |
| 144 | 2.32 | 42.4 | 54 |
| 145 | 1.27 | 37 | 43.35 |
| 146 | 2.34 | 44 | 55.7 |
| 147 | 1.51 | 33.3 | 40.85 |
| 148 | 1.61 | 39.7 | 47.75 |
| 149 | 1.27 | 37.8 | 44.15 |
| 150 | 1.2 | 41.3 | 47.3 |
| 151 | 3.02 | 42.7 | 57.8 |
| 152 | 1.54 | 40.3 | 48 |
| 153 | 1.1 | 42 | 47.5 |
| 154 | 1.25 | 34.2 | 40.45 |
| 155 | 2.07 | 34 | 44.35 |
| 156 | 0.82 | 35 | 39.1 |
| 157 | 1.99 | 34 | 43.95 |
| 158 | 1.47 | 32 | 39.35 |
| 159 | 1.98 | 34 | 43.9 |
| 160 | 0.93 | 24.9 | 29.55 |
| 161 | 1.74 | 32.4 | 41.1 |
| 162 | 2.01 | 42 | 52.05 |
| 163 | 2.65 | 37 | 50.25 |
| 164 | 0.99 | 34.2 | 39.15 |
| 165 | 2.51 | 39.7 | 52.25 |
| 166 | 0.88 | 40.2 | 44.6 |
| 167 | 4.98 | 42.5 | 67.4 |
| 168 | 1.87 | 36.1 | 45.45 |
| 169 | 0.89 | 44.1 | 48.55 |
| 170 | 0.9 | 35.3 | 39.8 |
| 171 | 1.23 | 38.1 | 44.25 |
| 172 | 1.15 | 42.7 | 48.45 |
| 173 | 1.81 | 29.8 | 38.85 |
| 174 | 1.61 | 41 | 49.05 |
| 175 | 1.01 | 32.7 | 37.75 |
| 176 | 2.95 | 33.8 | 48.55 |
| 177 | 1.53 | 45.1 | 52.75 |
| 178 | 1.32 | 30.5 | 37.1 |
| 179 | 1.27 | 43.6 | 49.95 |
| 180 | 1.5 | 43.3 | 50.8 |
| 181 | 2.38 | 35.4 | 47.3 |
| 182 | 1.77 | 41.6 | 50.45 |
| 183 | 1.3 | 34.4 | 40.9 |
| 184 | 1.45 | 45.6 | 52.85 |
| 185 | 2.25 | 36.7 | 47.95 |
| 186 | 0.65 | 31.6 | 34.85 |
| 187 | 0.7 | 37.5 | 41.03 |
| 188 | 2.75 | 29.3 | 43.05 |
| 189 | 2.01 | 34.2 | 44.25 |
| 190 | 1.7 | 40 | 48.5 |
| 191 | 1.27 | 40 | 46.35 |
| 192 | 0.51 | 38.7 | 41.23 |
| 193 | 0.58 | 44.2 | 47.1 |
| 194 | 3.46 | 33 | 50.3 |
| 195 | 2.41 | 41.1 | 53.15 |
| 196 | 1.46 | 50.1 | 57.4 |
| 197 | 1.41 | 46.9 | 53.95 |
| 198 | 2.02 | 45.7 | 55.8 |
| 199 | 1.75 | 40.9 | 49.65 |
| 200 | 1.56 | 37 | 44.8 |
| 201 | 1.93 | 36.2 | 45.85 |
| 202 | 1.05 | 33.9 | 39.15 |
| 203 | 0.66 | 30.1 | 33.4 |
| 204 | 1.06 | 28.9 | 34.2 |
| 205 | 1.39 | 43 | 49.95 |
| 206 | 1.06 | 38 | 43.3 |
| 207 | 1.36 | 40.1 | 46.9 |
| 208 | 1.51 | 38 | 45.55 |
| 209 | 2.49 | 42.7 | 55.15 |
| 210 | 1.87 | 33.9 | 43.25 |
| 211 | 1.44 | 40.7 | 47.9 |
| 212 | 2.36 | 37.8 | 49.6 |
| 213 | 0.83 | 40 | 44.15 |
| 214 | 1.5 | 39.7 | 47.2 |
| 215 | 2.4 | 43 | 55 |
| 216 | 1.48 | 34 | 41.4 |
| 217 | 0.7 | 31.6 | 35.1 |
| 218 | 0.79 | 27 | 30.95 |
| 219 | 1.75 | 31 | 39.75 |
| 220 | 1.89 | 39.6 | 49.05 |
| 221 | 1.35 | 33 | 39.75 |
| 222 | 3.2 | 38.4 | 54.42 |
| 223 | 1.68 | 29.6 | 38 |
| 224 | 1.33 | 30.5 | 37.15 |
| 225 | 1.35 | 31.4 | 38.15 |
| 226 | 2.67 | 42.9 | 56.25 |
| 227 | 1.87 | 33.9 | 43.25 |
| 228 | 2.3 | 38 | 49.5 |
| 229 | 0.65 | 37 | 40.25 |
| 230 | 1.45 | 38.8 | 46.05 |
| 231 | 2.41 | 35.5 | 47.55 |
| 232 | 2.36 | 37.3 | 49.1 |
| 233 | 1.6 | 40.1 | 48.1 |
| 234 | 1.88 | 43.8 | 53.2 |
| 235 | 1.4 | 39.7 | 46.7 |
